# Antibiotic resistant bacteria: A bibliometric review of literature

**DOI:** 10.3389/fpubh.2022.1002015

**Published:** 2022-11-17

**Authors:** Guojun Sun, Qian Zhang, Zuojun Dong, Dashun Dong, Hui Fang, Chaojun Wang, Yichen Dong, Jiezhou Wu, Xuanzhe Tan, Peiyao Zhu, Yuehua Wan

**Affiliations:** ^1^College of Pharmaceutical Science, Zhejiang University of Technology, Hangzhou, China; ^2^Institute of Information Resource, Zhejiang University of Technology, Hangzhou, China; ^3^Hangzhou Aeronautical Sanatorium for Special Service of Chinese Air Force, Hangzhou, China; ^4^Department of Chinese Medicine, Macau University of Science and Technology, Taipa, Macau SAR, China

**Keywords:** antibiotic resistant bacteria, antibiotic resistance, antibiotics, bibliometrics, keyword analysis

## Abstract

Antibiotic-resistant bacteria (ARB) are a serious threat to the health of people and the ecological environment. With this problem becoming more and more serious, more countries made research on the ARB, and the research number has been sharply increased particularly over the past decade. Therefore, it is quite necessary to globally retrace relevant researches on the ARB published from 2010 to 2020. This will help researchers to understand the current research situation, research trends and research hotspots in this field. This paper uses bibliometrics to examine publications in the field of ARB from 2010 to 2020 that were retrieved from the Web of Science (WOS). Our study performed a statistical analysis of the countries, institutions, journals, authors, research areas, author keywords, Essential Science Indicators (ESI) highly cited papers, and ESI hotspots papers to provide an overview of the ARB field as well as research trends, research hotspots, and future research directions in the field. The results showed that the number of related studies is increasing year by year; the USA is most published in the field of ARB; China is the most active in this field in the recent years; the Chinese Acad Sci published the most articles; Sci. Total Environ. published the greatest number of articles; CM Manaia has the most contributions; Environmental Sciences and Ecology is the most popular research area; and “antibiotic resistance,” “antibiotics,” and “antibiotic resistance genes” were the most frequently occurring author keywords. A citation analysis showed that aquatic environment-related antibiotic resistance is a key research area in this field, while antimicrobial nanomaterial-related research is a recent popular topic.

## Introduction

Antibiotic-resistant bacteria are resistant to both natural and synthetic antibiotics (1) and thus have become a health concern worldwide. Multi-drug resistant bacteria (MDRB) with stronger resistance can be resistant to 3 or more antibiotics in clinic (2–5). Bacteria can develop intrinsic resistance to certain antibiotics, but can also acquire resistance to antibiotics (6). Among them, the path for bacteria to acquire or development antibiotic resistance which roots in the irrational usage of antibiotics is to prevent antibiotics from entering target, change the antibiotic targets and inactivate antibiotics (6–9). The irrational usage of antibiotics can lead to the prolonged exposure of bacteria to sublethal concentrations of antibiotics which is a key to the resistance selection (10, 11). Because antibiotics with sublethal concentrations cannot kill bacteria, but can affect the frequency of mutations, horizontal gene transfer (HGT) and gene recombination of bacteria, and have a chance to enrich existing low-level resistant mutations or improve the level of drug resistance mutation. The spread of antibiotic resistance among different bacterial populations is achieved through HGT (12). HGT refers to the transfer of antibiotic resistance genes (ARGs) between bacteria by transformation, transduction, and conjugation with the help of plasmids, integrons, transposons and so on (13). A large number of bacterial species are resistant to macrolides, sulfonamides, tetracyclines, and other antibiotics in the biological systems (14). Antibiotic has become synonymous with “antibacterial drug” in some degree, therefore, in this review antibiotic has been used.

Antibiotics are not completely metabolized in the human body, and some are excreted into the sewage with urine and feces in prototype (10). As the sewage treatment process has created a potential environment suitable for the development and spread of antibiotic resistance, such as high bacterial density, pressure caused by pollutants such as heavy metals and antibiotics, etc. Therefore, the discharge of treated sewage gives rise to a large number of ARB and ARGs in the surrounding ecological environment (e.g., aquatic system and soil) (12, 15–21). Moreover, the proportion of antibiotic resistance in chickens, pigs, and wild animals has also increased greatly (22), thus causing a serious burden of infection to human beings (23–25), and greatly affecting the ecological environment (26). Humans can be infected with ARB in different ways. For example, ARB in communities and medical settings can be transmitted through person-to-person contact (27). Healthcare associated infections (HAIs) are infections caused to patients by invasive devices or surgical procedures, such as catheter-associated urinary tract infections, surgical site infections, and ventilator-associated pneumonia (28), which are also common infections with ARB. Antibiotic-resistant bacteria can also be transmitted to people through the environment. For example, driven by hydrological processes such as runoff and infiltration, the treated sewage enters the sources of drinking water, such as surface water and groundwater, after being discharged into the environment, resulting in ARB and ARGs in the drinking water sources (29). However, conventional drinking water treatment is mainly designed to remove contaminants such as heavy metals, solid particles and pathogenic microorganisms, rather than to remove ARB, which may even promote the transmission of ARB from the environment to humans (29, 30). Soil may lead to the transfer of resistance determinants from the environment or zoonotic bacteria to humans (31). When the ARB infect the human body, it can transfer to the human pathogenic bacteria. Once the pathogenic bacteria develop resistance, it is harder to control and treat bacterial infections (29). For example, antibiotic resistance may lead to increased virulence and pathogenicity, increased morbidity and mortality, longer hospital stays, and reduced availability of antibiotics (32, 33). According to the WHO, 10 million people may die from ARB infections every year by 2050. In 2010, the Infectious Diseases Society of America started the “10 × ‘20 Initiative”, with the goal of developing 10 effective antibacterial medications by 2020 (34). The WHO published a priority list in 2018 to guide the creation of new antibiotics (35). However, the rate of new antibiotic research and development is surprisingly slow (36). Very few new structural classes of antibiotics have been introduced since 2000 (37, 38), e.g., cyclic lipopeptide (daptomycin) (39, 40), oxazolidinone (linezolid) (41), etc. Yet more and more bacteria are resistant to many antibiotics used clinically (42, 43). We are no longer confident in the face of more and more bacterial infections (6). Therefore, new antimicrobial strategies are particularly important (44). In the early stage, it was mainly treated in combination with other antibiotics, such as streptomycin and penicillin. The combination of antibiotics has a synergistic effect, which not only has better efficacy than a single drug, but also can inhibit the drug resistance selection of a single drug (45, 46). With the development of multi-drug resistant bacteria, antibiotic substitutes (47) such as phage therapy (48–50), nanomaterials (51–54), bacteriocins (55), antibodies, and probiotics (56) have been attracted more attention.

The earliest monographic study in the field of ARB was published in 1990, and it provided an initial description of the antibiotic resistance mechanism (57). Findings over the subsequent decade included the identification of ARB in aquaculture for the first time (58–60), which was based on irrational antibiotic use in aquaculture (61). In addition, preliminary studies on the spread of ARB (62, 63), doctors' prescriptions (64) as well as phage therapy (65) were performed. During the period from 2000 to 2009, the findings focused on the fact that ARB and ARGs were discovered in wastewater and drinking water (66, 67). Antibiotic resistance (68–70), nanorods (71), phage therapy (72), and rational antibiotic use interventions (73) were further studied. In the last decade, with the development and application of polymerase chain reaction (PCR) assays (74, 75) and metagenomic analysis (76–79), the abundance of multiple ARGs could be identified. Consequently, ARB and ARGs were detected in aquatic systems, such as wastewater (80, 81), rivers (82–85), lakes (86), seawater (87), drinking water (88), reclaimed wastewater (89), and aquaculture (90), as well as animal husbandry (91, 92), compost (93), soil (94, 95), and vegetables (96, 97). For the sake of preventing the spread of ARB and ARGs in the environment and mitigating the damage to humans, animals and the ecological environment, an increasing number of researchers have devoted themselves to finding solutions to this difficult problem. Hence, a large number of processes for removing antibiotics, ARB and ARGs from wastewater have emerged, including chlorination (98, 99), ultraviolet (UV) (100, 101), advanced oxidation processes (AOPs) (102, 103), ozonation (104), solar photo-Fenton (105–107), photocatalytic oxidation (108, 109), constructed wetlands (CWs) (110), and membrane bioreactors (MBRs) (111). Even though studies on ARB and ARGs in wastewater and drinking water were carried out from 2000 to 2009 and from 2010 to 2020, the research content from 2010 to 2020 was more focused. Since the comparison and analysis of ARB and ARGs were generally conducted from 2000 to 2009, most of the samples collected in this stage were from source water, effluent from sewage treatment plants or rivers, while the research from 2010 to 2020 targeted more on the sewage treatment process. The samples collected in this stage may come from different treatment steps in the sewage process. For example, it may come from sand filtration and peracetic acid treatment (112) or various sewage treatment methods, e.g., chlorination (99), ozone (104), etc. Moreover, the detection technologies employed during 2010–2020 are more efficient, such as high-throughput sequencing technology (14).

ARB is highly interrelated to human and ecological health, and there has been more extensive previous studies in this field, the priority list of ARB (35), ARB persistence (113), the challenge of ARB in the food industry (114), the antibiotic resistance profiles (19, 22) antimicrobial strategies (115–117) and antibiotics discovery (36). ARB are a serious threat to the health of people and the ecological environment. With this problem becoming more and more serious, more countries made research on the ARB, and the research number has been sharply increased particularly over the past decade. Therefore, it is quite necessary to globally retrace relevant researches on the ARB in recent 10 years. This will help researchers to understand the current research situation, research trends and research hotspots in this field.

Bibliometric analysis is an effective method for quantitatively assessing academic papers and can be used to investigate the evolution of certain fields, and the results can provide an overview of a certain field as well as research trends, hot topics, distribution of research power and future research directions (118–122). The advantage of bibliometric is that it is not limited by geography, allowing data to be collected by country in a particular area to analyze research globally (123). In addition, specific data analysis software can process the results of bibliometric analyses and present them in a more three-dimensional form (124–127). Therefore, bibliometric analyses have been applied to many fields, such as medicine (128–130), chemistry (131), psychology (132), computer science (133, 134), and robotics (120). In addition, bibliometrics is also widely applied to the aspect of research method, for example, the publications related to such research methods as TOPSIS (135), Analytic Hierarchy Process (136), and ordered weighted averaging operator (137) can also make knowledge recreation by bibliometrics.

To our knowledge bibliometric analysis of publications in the field of ARB has been conducted, but related studies only focused on antibiotics in soil (138) and ARGs (139). Since the study of ARB is multifaceted, such as generation (6), impact (23), control (140), and treatment (55) of ARB, and so on, a comprehensive analysis of ARB research from a bibliometric perspective remains necessary. The goal of this paper is to apply a bibliometric approach to review the leading countries, institutions, authors, and journals, research areas, national and institutional collaborations, author keywords, and ESI highly cited and hot papers to provide research situation, research trends and research hotspots in the field of ARB between 2010 and 2020 globally and then propose future research directions.

## Materials and methods

A bibliometric analysis of publications in the field of ARB published between 2010 and 2020 is presented in this paper. Data were obtained from the Science Citation Index Expanded database (SCI-E) and Social Sciences Citation Index database (SSCI). Scopus, Pubmed and Google Academic indeed cover more publications than Web of Science. However, the publications included into the core complications of WOS generally receive higher recognition and it is the most widely accepted database for analysis of science publications (141). Therefore, WOS was chosen as the data source for this study. First, the subject field was set to “antibiotic resistant bacteria”, the date range was set to 2010-01-01 to 2020-12-31, and the document type was set to “article” and “review” for the search. The corresponding country, institution, journal, author, author keywords, and research area of publications meeting the search criteria are listed. The same data were extracted from ESI highly cited and hot papers. Then, the Derwent Data Analyzer (DDA10.0 build 27,330, Search Technology Inc., Norcross, GA, USA), which is a tool for data cleaning, mining and visual processing, was used to clean the derived data.

Although ARB is an acronym for antibiotic resistant bacteria, it was not included in the search formula because the acronym is used in other fields. Antimicrobial include antibiotics, however it was not included in the search formula, because antimicrobial is not only effective against ARB, it is also effective against mycoplasma, chlamydia, viruses, etc. Articles from Scotland, Wales, England, and Northern Ireland are included as papers from the UK. Each journal's impact factor is derived from the 2020 JCR. Not all relevant articles were included in this analysis, and those that did not match the search rules were excluded. In this review DDA has been used to make matrix map, cluster map, bubble chart and cross-correlation plot. Since publications are time-sensitive, this paper only analyzed the literature published from 2010 to 2020.

## Results

**Figure 1:**
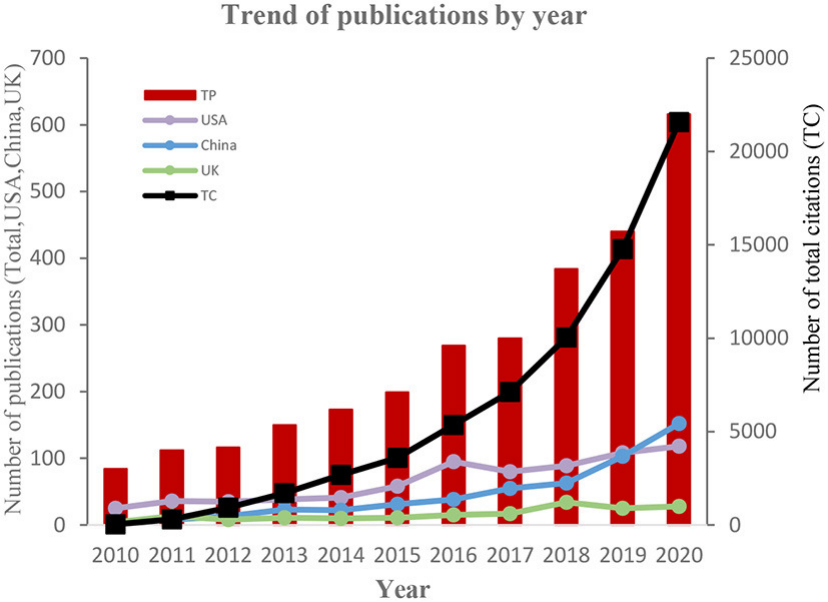
Trends in the number of published articles related to ARB by year. TP, total papers; TC, total citations.

From 2010 to 2020, 2,823 papers in the ARB field were published by authors in 116 countries, including 99 ESI highly cited papers and 3 ESI hot papers. These publications can be divided into 11 languages, including 2,793 in English (98.94%), 10 in German (0.35%), 6 in Spanish (0.213%), 3 in French and Polish (0.106%), 2 in Hungarian and Portuguese (0.071%), and 1 in Chinese, Dutch, Italian and Turkish (0.035%). The growth trend of articles related to the ARB field from 2010 to 2020 was described (Figure 1). During this period, the number of articles published in this field increased by more than seven-fold, with the number of articles published from 2018 to 2020 increasing significantly. This finding indicates that ARB has attracted increasing concern year by year, and it also shows that the impact of ARB on human beings is increasing.

### Contribution of leading countries/regions

**Figure 2:**
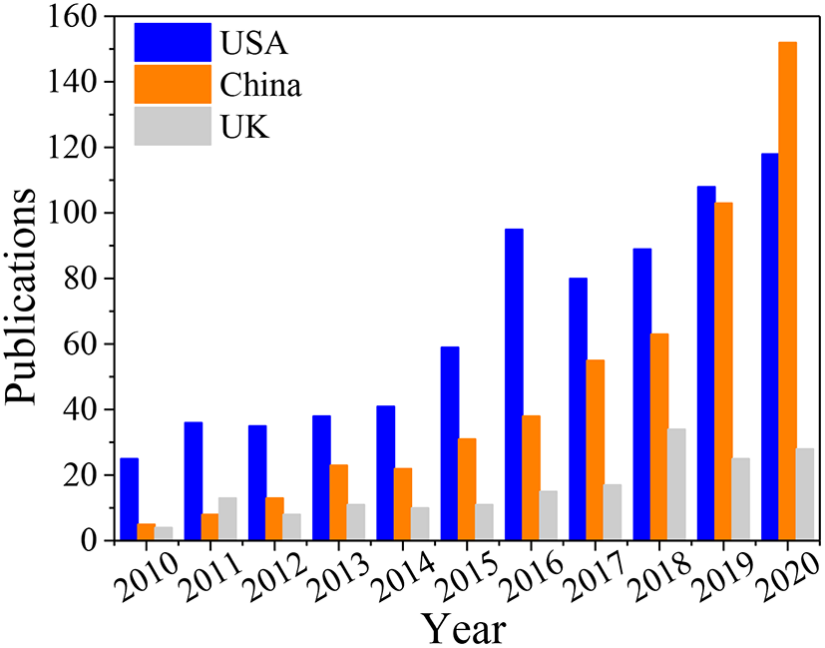
Number of ARB-related publications per year from 2010 to 2020 in the USA, China and the UK.

The top 20 countries in terms of total quantity of publications in the ARB field between 2010 and 2020 were identified (Table 1). The USA is the country with the most publications in this field, followed by China and the UK, whose publications account for 25.61, 18.17, and 6.23% of the total publications, respectively. The same result can be seen in the ranking of total citations; that is, the USA is first, followed by China and the UK. Figure 2 shows the number of ARB-related publications per year from 2010 to 2020 in the USA, China and the UK. It can be seen that China issued very few publications from 2010 to 2013, less than the UK and the USA, while in 2019 the number of publications in China rose significantly. In 2020 China has already surpassed the USA in the number of relevant publications. This indicates that China is considerably more active in this research field during recent years. It is likely related to the large population in China, the high prevalence of antibiotic abuse (142), the relevant policies (143, 144) and higher scientific research fund support (145). Among the top 20 countries, 11 countries were in Europe, 5 countries were in Asia, and 4 countries were in the Americas, which shows that ARB have attracted global attention.

**Table 1:** The top 20 most productive countries/regions in the ARB field.

**Rank**	**Country**	**TP**	**TC**	**h-index**	**ACPP**	**nCC**	**SP (%)**
1	USA	723	27,927	78	38.63	67	40.11
2	China	513	16,157	64	31.5	43	32.75
3	UK	176	10,977	43	62.37	64	69.32
4	Germany	168	10,219	43	60.83	57	61.90
5	Italy	140	8,384	36	59.89	48	52.14
6	Spain	139	6,584	38	47.37	52	64.75
7	India	134	2,941	30	21.95	43	34.33
8	South Korea	121	3,168	32	26.18	35	37.19
9	Sweden	104	7,246	33	69.67	48	59.62
10	Canada	101	6,148	34	60.87	46	64.36
11	France	99	7,044	32	71.15	55	64.65
12	Japan	99	2,317	24	23.4	32	39.39
13	Australia	98	6,120	36	62.45	47	75.51
14	Portugal	96	6,808	34	70.92	47	44.79
15	Netherlands	79	5,778	32	73.14	45	62.03
16	Poland	79	3,298	27	41.75	39	34.18
17	Brazil	77	1,509	22	19.6	19	41.56
18	Switzerland	67	4,386	28	65.46	39	59.70
19	Iran	55	969	18	17.62	11	18.18
20	Turkey	49	876	15	17.88	17	28.57

TP, total papers; TC, total citations; ACPP, average citations per publication; nCC, number of cooperative countries; SP, Share of publications.

### Cooperation of leading countries/regions

**Figure 3:**
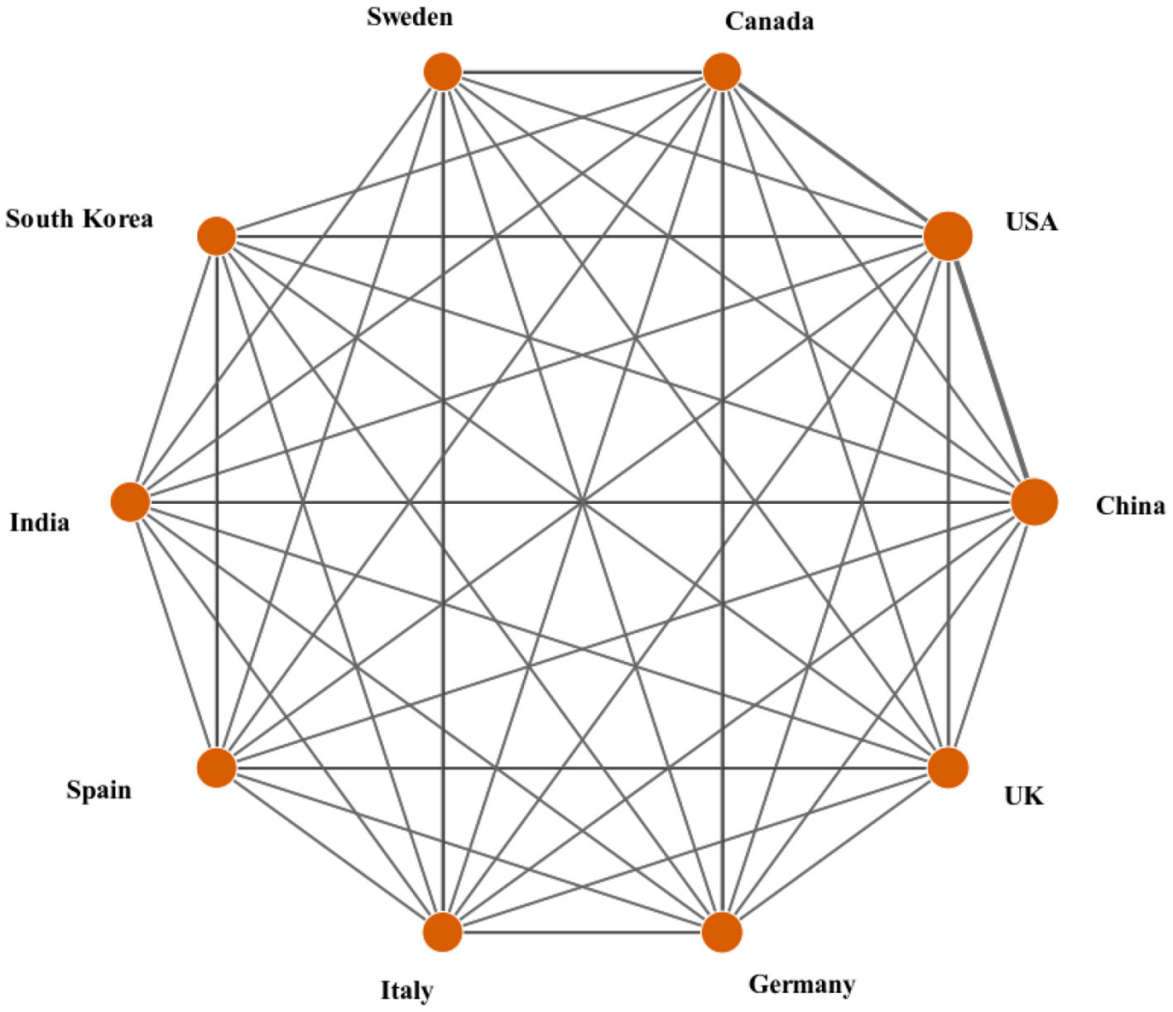
Collaboration matrix map among the top 10 productive countries/regions.

The most impactful science comes from international collaboration (146), which is based on the flow and integration of knowledge. Different countries/regions may have different emphases when studying ARB, although resource complementarity and continuous innovation impulses can be achieved by collaboration. International collaborative publications are joint papers written by scholars from multiple countries. The number of cooperative countries (nCC) refers to how many countries a country has cooperated with in a certain field. It can be concluded from Table 1 that among all countries, the USA, the UK, Germany, Spain and France have more cooperation with other countries. To better understand the current state of international collaboration in the ARB field, a network graph between the top 10 countries/regions was created using the DDA software (Figure 3). The circle size symbolizes the countries' contributions, the lines connecting the circles indicate cooperation between countries, and the thickness of the lines indicates the number of collaborative publications. It can be seen from Figure 3 that almost all of the top 10 countries in publications have ever cooperated with each other. The line between the USA and China is the thickest, which indicates that the number of cooperative publications between the USA and China is the largest in this field, followed by the number of cooperative publications between the USA and Canada.

### Contribution of leading institutions

Statistics on the contributions of leading institutions can help us identify the most authoritative professional institutions in the ARB field. There are 3,430 institutions involved in ARB research, and the top 20 are summarized (Table 2). Among these 20 institutions, there are 40% institutions in Europe and Asia, respectively, while the majority of those in Asia are from China. Although the Chinese Acad Sci has published a large amount of articles, the total citations and average citations per paper are not the highest. Although several European institutes do not have a large number of publications, such as Univ Catolica Portuguesa and Univ Cyprus, the quality of articles is relatively high, which can be seen from their high total citations and average citations per paper.

**Table 2:** The top 20 most productive institutions in the ARB field during 2010–2020.

**Rank**	**Institutions**	**TP**	**TC**	**ACPP**	**h-index**	**TPR (%)**	**Country/Region**
1	Chinese Acad Sci	77	3,443	44.71	31	2.728	China
2	Univ Porto	37	2,046	55.30	21	1.311	Portuguese
3	USDA ARS	36	986	27.39	17	1.276	USA
4	Univ Catolica Portuguesa	34	4,239	124.68	24	1.204	Portuguese
5	Univ Chinese Acad Sci	32	1,455	45.47	18	1.134	China
6	Univ Salerno	28	2,732	97.57	19	0.991	Italy
7	Tsinghua Univ	27	1,355	50.19	18	0.956	China
8	Zhejiang Univ	26	450	17.31	11	0.921	China
9	Karolinska Inst	25	704	28.16	13	0.886	Sweden
10	Univ Gothenburg	24	2,070	86.25	17	0.850	Sweden
11	Univ Queensland	24	1,091	45.46	15	0.850	Australia
12	Univ Copenhagen	23	1,299	56.48	13	0.815	Denmark
13	Uppsala Univ	23	1,590	69.13	15	0.815	Sweden
14	Natl Univ Singapore	22	1,047	47.59	16	0.779	Singapore
15	Tongji Univ	22	905	41.14	15	0.779	China
16	Univ Cyprus	22	3,609	167.73	17	0.779	Cyprus
17	Univ Maryland	22	796	35.91	14	0.779	USA
18	Sun Yat Sen Univ	21	576	27.43	11	0.743	China
19	Univ Minnesota	21	1,275	60.71	12	0.743	USA
20	Nankai Univ	20	1,154	57.70	13	0.708	China

TP, total papers; TC, total citations; ACPP, average citations per publication; TPR, the percentage of articles of institutions in total publications.

**Figure 4:**
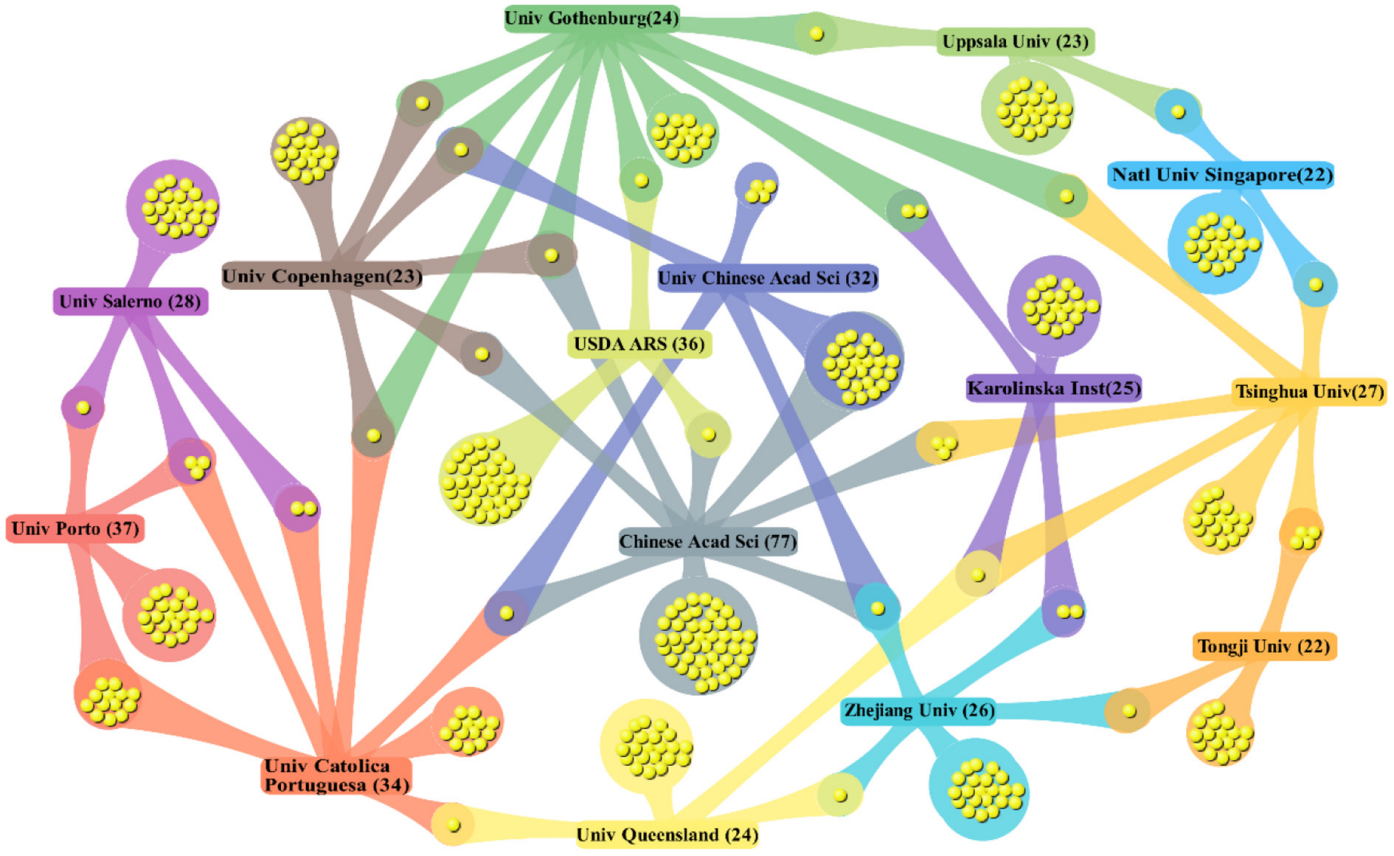
DDA cluster map on cooperation of the top 15 institutions.

The output and quality of scientific research were positively correlated with the degree of international collaboration (147). A cluster map of the collaboration among the top 15 institutions was created with DDA software (Figure 4). Obviously, Gothenburg University, the Chinese Acad Sci and Tsinghua University showed the most extensive collaborations with other institutions in the ARB field. In addition, the USDA ARS, Karolinska Inst and Univ Queensland have a greater number of collaborations with institutions in different countries; thus, their degree of internationalization was high. The collaborations between the Chinese Acad Sci and Univ Chinese Acad Sci and between Univ Porto and Univ Catolica Portuguesa were the most frequent. Institutions in European countries were more closely connected with those in neighboring countries/regions, which was similar to that in Asia, possibly because of factors such as institutional relationships and geographical proximity.

### Contribution of leading journals

The collation of published journals revealed that a total of 983 journals published ARB-related research from 2010 to 2020. The top 30 journals by the number of articles are displayed (Table 3). These 30 journals have published a total of 911 articles on ARB, accounting for 45.86% of the total literature. Forty-three percent of these journals were related to the environment, 20% were related to microbiology, 13% were related to medicine, 10% were related to engineering technology, and 3% was related to materials and chemistry each. The breadth of disciplines involved reflects that ARB represent an interdisciplinary research field.

**Table 3:** Top 30 journals publishing papers in ARB research.

**Rank**	**Journal title**	**TP**	**TC**	**ACPP**	**IF**	**TPR (%)**
1	Sci. Total Environ.	110	5,546	50.42	7.963	3.897
2	Front. Microbiol.	79	2,656	33.62	5.64	2.798
3	PLoS One	69	1,780	25.8	3.24	2.444
4	Water Res.	65	4,684	72.06	11.236	2.303
5	Chemosphere	40	1,799	44.98	7.086	1.417
6	Sci Rep	40	591	14.78	4.38	1.417
7	Antibiotics-Basel	36	391	10.86	4.639	1.275
8	Environ. Sci. Pollut. Res.	35	936	26.74	4.223	1.24
9	J. Hazard. Mater.	30	992	33.07	10.588	1.063
10	Antimicrob. Agents Chemother.	29	984	33.93	5.191	1.027
11	Environ. Sci. Technol.	28	2,215	79.11	9.028	0.992
12	Int. J. Environ. Res. Public Health	28	772	27.57	3.39	0.992
13	Environ. Pollut.	27	956	35.41	8.071	0.956
14	mBio	26	1,154	44.38	7.867	0.921
15	Environ. Int.	25	1,177	47.08	9.621	0.886
16	Appl. Environ. Microbiol.	22	997	45.32	4.813	0.779
17	ACS Appl. Mater. Interfaces	19	636	33.47	9.229	0.673
18	Appl. Microbiol. Biotechnol.	19	545	28.68	4.813	0.673
19	Microb. Drug Resist.	18	228	12.67	3.431	0.638
20	Clin. Infect. Dis.	17	1,844	108.47	9.079	0.602
21	Environ. Monit. Assess.	17	409	24.06	2.513	0.602
22	J. Antimicrob. Chemother.	17	476	28	5.79	0.602
23	Microorganisms	16	118	7.38	4.128	0.567
24	Water Sci. Technol.	16	168	10.5	1.915	0.567
25	Chem. Eng. J.	15	500	33.33	13.273	0.531
26	J. Environ. Qual.	14	395	28.21	2.751	0.496
27	J. Food Prot.	14	196	14	2.077	0.496
28	Molecules	14	408	29.14	4.412	0.496
29	Ecotox. Environ. Safe.	13	670	51.54	6.291	0.461
30	Int. J. Nanomed.	13	370	28.46	6.4	0.461

TP, total papers; TC, total citations; ACPP, average citations per publication; IF, Impact Factor 2020; TPR, the percentage of articles of journals in total publications.

### Contribution of leading authors

Statistics on leading authors can help us understand the top experts in the ARB field. A total of 13,966 authors were counted among 2,823 articles, of which 12,086 authors only published one article, 337 authors published three articles, and 15 authors published 10 or more articles. The top 20 authors in the number of articles and their institutions are summarized (Table 4). These authors published 245 articles, accounting for 8.67% of all articles. CM Manaia has published the most articles in this field and made important contributions to the presence and removal process of antibiotics, ARB and ARG in wastewater and antibiotic resistance in the environment. L Rizzo mainly studied sewage treatment processes, such as photocatalysis and UV. In addition to the study of sewage treatment processes, D Fatta-Kassinos also contributed to the reuse of wastewater.

**Table 4:** Contribution of the top 20 authors in ARB research.

**Rank**	**Author**	**TP**	**TAR**	**TC**	**ACPP**	**h-index**	**Institution (current), country/region**
1	Manaia, CM (148–150)	33	20	3,296	99.88	24	Univ Catolica Portuguesa, Portugal
2	Rizzo, L (151–153)	26	22	2,704	104	19	Univ Salerno, Italy
3	Fatta-Kassinos, D (154–156)	21	9	3,754	178.76	16	Univ Cyprus, Cyprus
4	Larsson, DGJ (157–159)	17	9	2,036	119.76	15	Univ Gothenburg, Sweden
5	Nunes, OC (160–162)	16	8	1,358	84.44	13	Univ Porto, Portugal
6	Pruden, A (140, 163, 164)	14	6	1,335	95.36	11	Virginia Tech, USA
7	Topp, E (165–167)	14	6	1,866	133.29	13	Agr and Agri Food Canada, Canada
8	Webster, TJ (168–170)	13	12	673	51.77	11	Northeastern Univ, USA
9	Schwartz, T (67, 171, 172)	12	2	2,687	223.92	11	Karlsruhe Inst Technol, Germany
10	Boopathy, R (173–175)	11	11	320	29.09	9	Nicholls State Univ, USA
11	Harnisz, M (176–178)	10	2	569	56.9	9	Univ Warmia and Mazury, Poland
12	Hong, PY (179–181)	10	9	347	34.7	9	King Abdullah Univ Sci and Technol, Arabia
13	Korzeniewska, E (182, 183)	10	6	569	56.9	9	Univ Warmia and Mazury, Poland
14	Pamer, EG (184–186)	10	6	1,481	148.1	8	Mem Sloan Kettering Canc Ctr,USA
15	Suzuki, S (187–189)	10	7	983	98.3	10	Ehime Univ, Japan
16	Ahn, J (190–192)	9	9	58	6.44	4	Kangwon Natl Univ, South Korea
17	Call, DR (193–195)	9	3	153	17	5	Washington State Univ, USA
18	Guo, MT (196–198)	9	9	312	34.67	9	Tongii Univ, China
19	Lundborg, CS (199–201)	9	1	402	44.67	8	Karolinska Inst, Sweden
20	Zhang, T (202–204)	9	3	1,223	135.89	8	Univ Hong Kong, China

TP, total papers; TAR, total number of articles for which they are responsible; TC, total citations; ACPP, average citations per publication.

### Contribution of leading research areas

Statistics on the research areas can help us grasp the shift of research emphasis in a specific field. There are 90 study areas associated with ARB, and the top 20 based on the number of articles are concluded (Table 5). The research areas of ARB are not only related to microorganisms, diseases, drugs, and chemistry but also related to the environment, engineering, agriculture, materials and oceanography, with the greatest number of publications related to the ecological environment. The top 5 areas accounted for 76.83% of all articles published, indicating that the environment, microbiology, engineering, drug and chemistry are the top research areas in the ARB field.

**Table 5:** Contribution of the top 20 research areas in ARB field.

**Rank**	**WOS research area**	**TP**	**TPR (%)**	**TC**	**ACPP**	**h-index**
1	Environmental sciences and ecology	697	24.69	28,631	41.08	83
2	Microbiology	545	19.306	23,188	42.55	71
3	Engineering	317	11.229	13,193	41.62	62
4	Pharmacology and pharmacy	314	11.123	8,238	26.24	46
5	Chemistry	296	10.45	9,583	32.38	51
6	Science and technology—other topics	279	9.883	8,932	32.01	55
7	Infectious diseases	261	9.246	11,324	43.39	42
8	Biotechnology and applied microbiology	210	7.439	5,330	25.38	41
9	Biochemistry and molecular biology	195	6.908	5,829	29.89	41
10	Water resources	159	5.632	6,370	40.06	39
11	Materials science	155	5.491	4,824	31.12	40
12	Public, environmental and occupational health	151	5.349	4,160	27.55	31
13	Immunology	109	3.861	4,722	43.32	33
14	Food science and technology	98	3.472	2,023	20.64	24
15	Veterinary sciences	82	2.905	995	12.13	18
16	Agriculture	76	2.692	1,978	26.03	24
17	General and internal medicine	71	2.515	3,320	46.76	25
18	Physics	51	1.807	1,490	29.22	21
19	Marine and freshwater biology	49	1.736	725	14.8	17
20	Biophysics	43	1.523	1,213	28.21	21

TP, total papers; TRP, percent of total articles in the field; TC, total citations; ACPP, average citations per publication.

**Figure 5:**
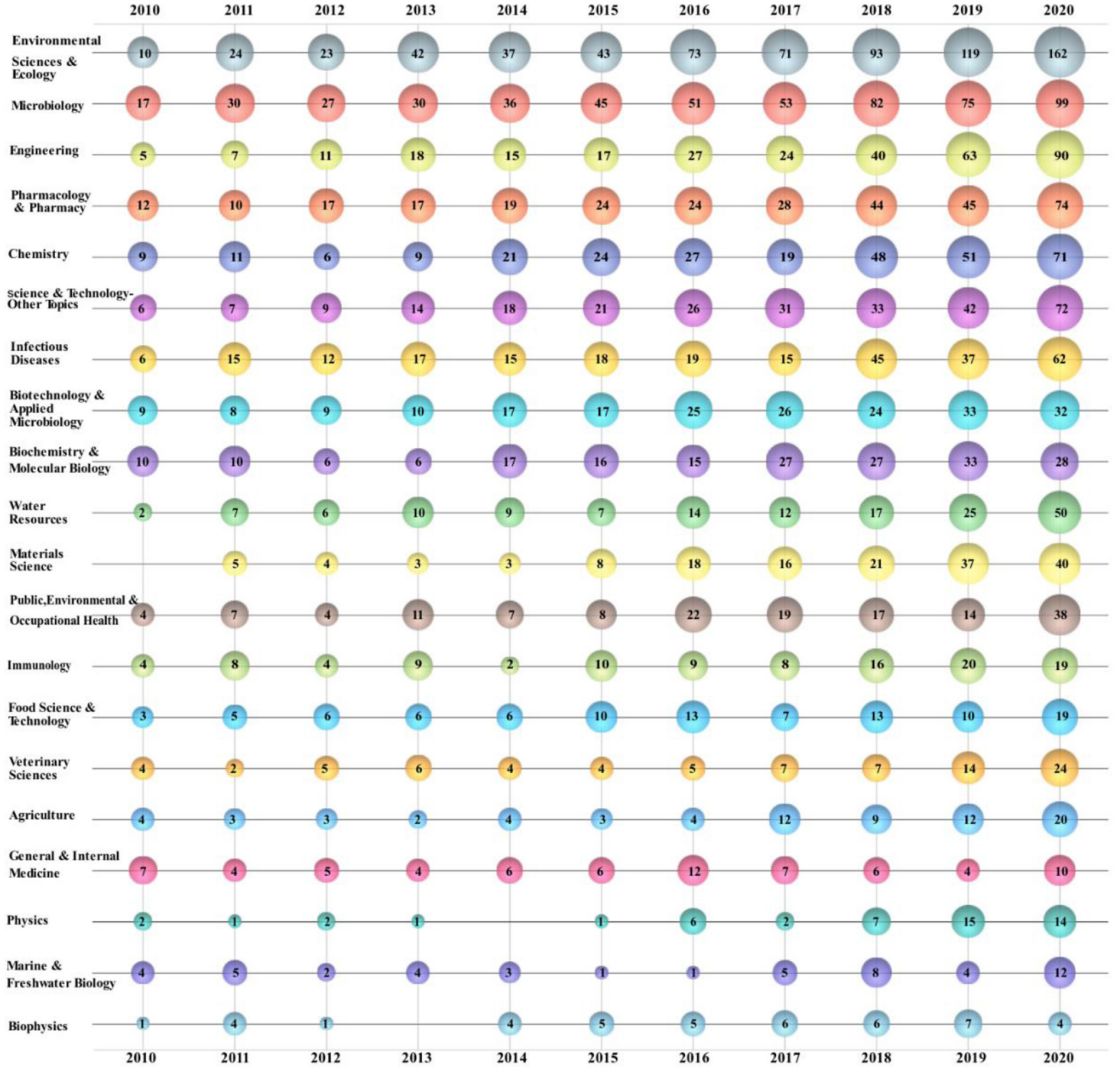
Bubble chart of top 20 ARB research areas.

The bubble chart can show the research trends and emphasis in a specific field more stereoscopically (205). A bubble chart is depicted to showing the top 20 ARB research areas (Figure 5). The numbers on the bubbles reflect the number of publications. “Environmental Sciences and Ecology” is the dominant research direction in the ARB field. From 2010 to 2020, the number of publications in this field increased and was the greatest overall, and it showed significant annual growth since 2017. “Microbiology” is also a research direction of increasing concern. The number of publications related to “Microbiology” every year is also on the rise, although a certain gap is observed. Compared with “Environmental Sciences and Ecology,” “Microbiology” received greater attention in the initial stage. Previously, the number of publications in the “Engineering” direction increased slowly but substantially between 2018 and 2020. The number of publications related to “Materials Science” was low in the initial phase but increased significantly after 2015, reaching a peak in the last 2 years.

### Analysis of author keywords

**Figure 6:**
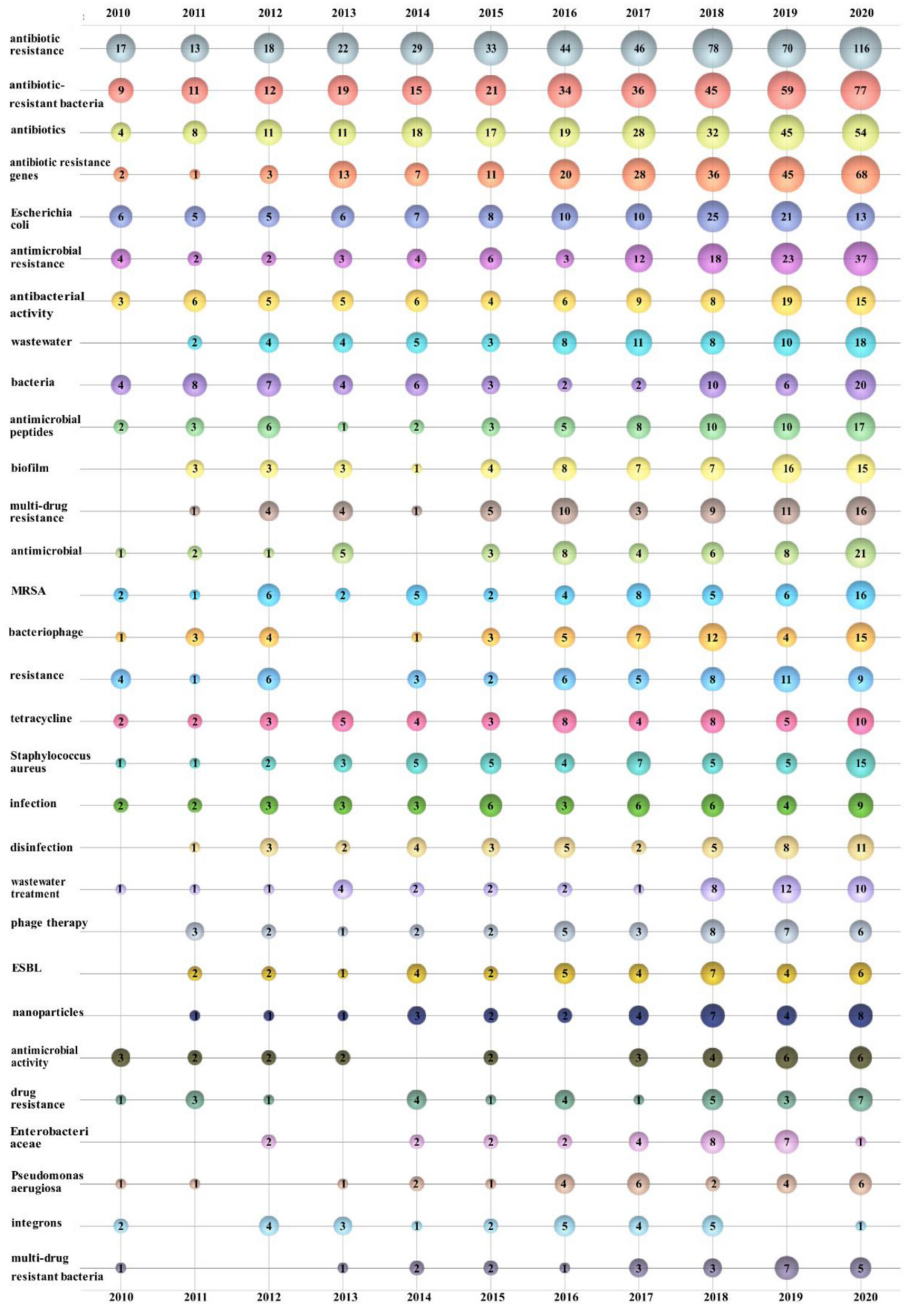
Bubble chart of the top 30 author keywords by year.

A keyword collection based on abundant academic findings in a research field over a long period of time can reveal the overall characteristics, developmental trends, and internal connections of such research. The top 30 author keywords from 2,823 publications were sorted and displayed in a bubble chart (206–209) in this study (Figure 6). The number on the bubble represents the times that the author keywords appeared in the corresponding year. In this paper, we combined author keywords with the same meaning through the DDA. Eventually, a total of 5,506 author keywords were obtained. Among them, 4,276 author keywords appeared only once, which accounted for 77.67%; 573 author keywords appeared twice, which accounted for 10.41%; and 6 author keywords appeared more than 100 times, which accounted for ~0.11%. Among them, “Antibiotic resistance,” “Antibiotic-resistant bacteria,” “Antibiotics,” and “Antibiotic resistance genes” had the highest appearance frequency. Much of the research on “Antibiotic resistance” has focused on the existence of “Antibiotics,” “Antibiotic-resistant bacteria,” and “Antibiotic resistance genes” in “Wastewater” and the environment and associated removal techniques. There are also many related studies on “Antibiotics,” “Antimicrobials,” “Antimicrobial peptides,” “MRSA,” “Nanoparticles,” and “Muti-drug resistant bacteria”.

**Figure 7:**
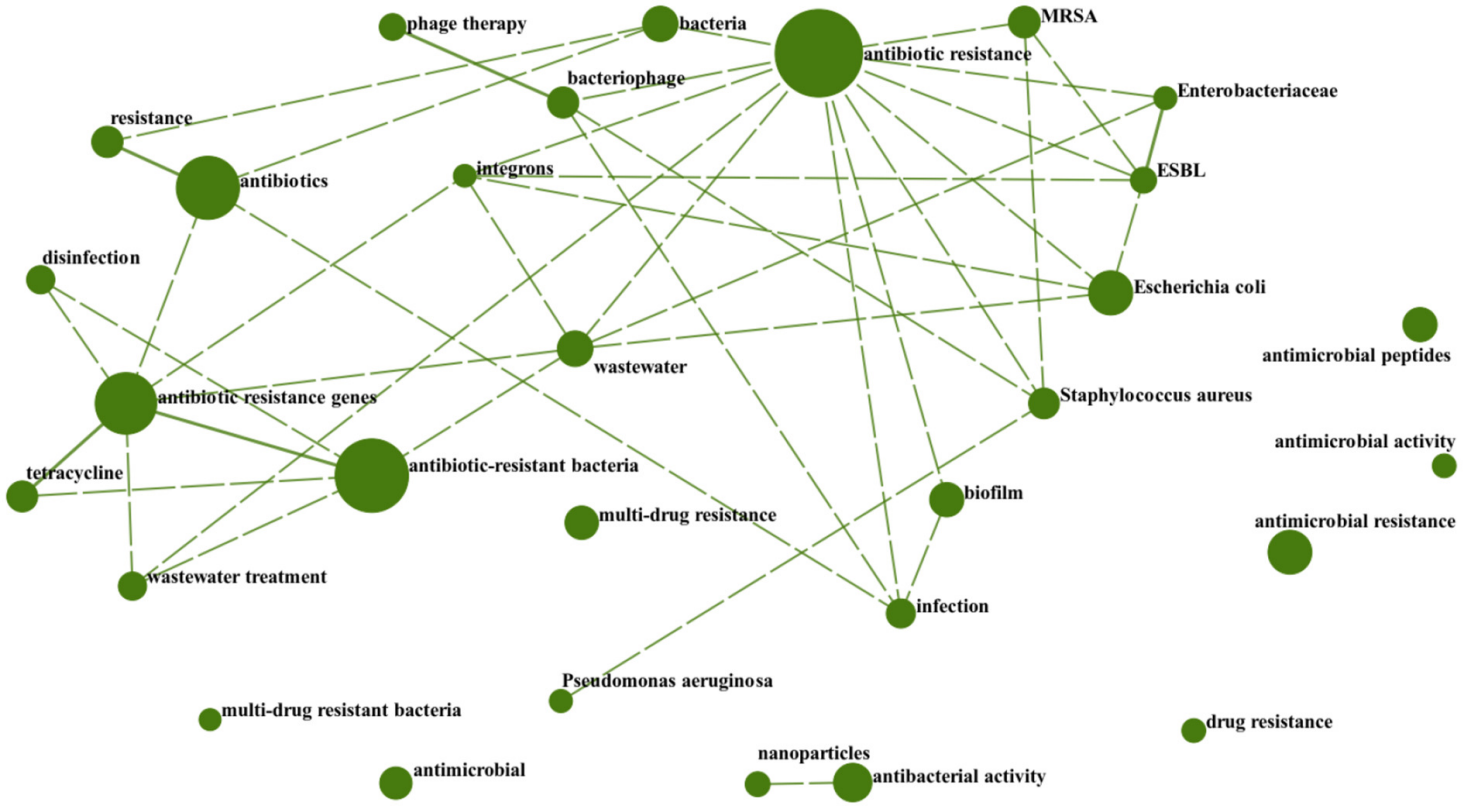
Cross-correlation graph of the top 30 author keywords.

The cross-correlation plot shows that two keywords occurred in one paper at the same time. Through the co-occurrence analysis of author keywords, the cross-connection between each author keywords can be better revealed. We designed a cross-correlation plot of the leading 30 author keywords by DDA (Figure 7). The size of the circle reflects how frequently the author keywords appear in total articles; the line connecting the two circles indicates that the two author keywords appear in the same article. The dashed line indicates a correlation between the two author keywords ranging from 0.25 to 0.5, and the solid line means 0.5–0.75. Undoubtedly, the author keywords with the highest frequency also correspond to the largest circles. We can also clearly discover that the author keywords appearing at the same time as “Antibiotic resistance” are the most, indicating that their research scope is wider. Among them, “Antibiotic-resistant bacteria” and “Antibiotic resistance genes,” “Resistance” and “Antibiotics,” “Phage therapy” and “Bacteriophage,” “Enterobacteriaceae” and “ESBL”, and “Antibiotic resistance genes” and “Tetracycline” are five pairs of closely related keywords, indicating that those two keywords had a high frequency of appearing simultaneously in an article.

### Analysis of ESI highly cited papers

The frequency of citations is a valuable metric for evaluating the impact of scientific papers (210, 211). The ESI highly cited papers refer to papers published in the last decade that presented a citation frequency ranked within the top 1% worldwide within the previous 2 months. Therefore, this paper adopts ESI highly cited papers to explore the hot topics of recent studies. The top 20 most cited papers in the ARB field from 2010 to 2020 are revealed (Table 6). Among these papers, the USA contributed 4 papers and the UK, Sweden and China each contributed 3 papers. Investigations to determine how antibiotic resistance develops in bacteria is the most frequently subject. Studies have focused on the main mechanisms of antibiotic resistance. The impact of ARB infection on humans is also of particular concern. In 2015, ARB infections were estimated to cause numerous deaths in Europe, with a high burden in infants and elderly individuals. Antibiotic resistance in wastewater has been a hot research topic in the last decade, with many studies related to Enterococcus and Escherichia coli. In addition, Acinetobacter baumannii, Pseudomonas aeruginosa (218), vancomycin-resistant Enterococcus (VRE) and methicillin-resistant Staphylococcus aureus (MRSA) (219) have a relatively large impact on humans and have recently received more attention. Guidelines for biological risk assessments of ARB production and transmission in the environment have also been controversial subjects in recent years because of their important roles in controlling antibiotic resistance in the environment.

**Table 6:** The top 20 most cited publications of ESI in ARB research field during 2010–2020.

**Rank**	**Corresponding** **authors**	**Title**	**TC**	**TCY**	**Publication** **year**	**Journal**	**Country/Region**
1	Piddock, LJV	Molecular mechanisms of antibiotic resistance (6)	1,578	263	2015	Nat. Rev. Microbiol.	UK
2	Tacconelli, E	Discovery, research, and development of new antibiotics: the WHO priority list of antibiotic-resistant bacteria and tuberculosis (35)	1,350	450	2018	Lancet Infect. Dis.	Germany
3	Rizzo, L	Urban wastewater treatment plants as hotspots for antibiotic resistant bacteria and genes spread into the environment: a review (18)	1,184	148	2013	Sci. Total Environ.	Italy
4	Manaia, CM	Tackling antibiotic resistance: the environmental framework (212)	896	149.33	2015	Nat. Rev. Microbiol.	Portugal
5	Cassini, A	Attributable deaths and disability-adjusted life-years caused by infections with antibiotic-resistant bacteria in the EU and the European Economic Area in 2015: a population-level modeling analysis (23)	814	407	2019	Lancet Infect. Dis.	Sweden
6	Cotter, PD	Bacteriocins—a viable alternative to antibiotics? (55)	804	100.5	2013	Nat. Rev. Microbiol.	Ireland
7	Fleming-Dutra, KE	Prevalence of inappropriate antibiotic prescriptions among US ambulatory care visits, 2010–2011 (9)	736	147.2	2016	JAMA-J. Am. Med. Assoc.	USA
8	Andersson, DI	Microbiological effects of sublethal levels of antibiotics (10)	744	106.29	2014	Nat. Rev. Microbiol.	Sweden
9	Gilmore, BF	Clinical relevance of the ESKAPE pathogens (31)	634	79.25	2013	Expert Rev. Anti-Infect. Ther.	UK
10	Guidos, RJ	10 × '20 Progress-development of new drugs active against gram-negative bacilli: an update from the Infectious Diseases Society of America (34)	539	67.38	2013	Clin. Infect. Dis.	USA
11	Xagoraraki, I	Release of antibiotic resistant bacteria and genes in the effluent and biosolids of five wastewater utilities in Michigan (213)	551	55.1	2011	Water Res.	USA
12	Zhu, YG	Antibiotic Resistome and its association with bacterial communities during sewage sludge composting (214)	484	80.67	2015	Environ. Sci. Technol.	China
13	Larsson, DGJ	Management options for reducing the release of antibiotics and antibiotic resistance genes to the environment (140)	434	54.25	2013	Environ. Health Perspect.	Sweden
14	Czaplewski, L	Alternatives to antibiotics-a pipeline portfolio review (47)	413	82.6	2016	Lancet Infect. Dis.	UK
15	Xagoraraki, I	Correlation of tetracycline and sulfonamide antibiotics with corresponding resistance genes and resistant bacteria in a conventional municipal wastewater treatment plant (215)	437	48.56	2012	Sci. Total Environ.	USA
16	Mao, DQ	Occurrence of sulfonamide and tetracycline-resistant bacteria and resistance genes in aquaculture environment (90)	442	49.11	2012	Water Res.	China
17	Grenni, P	Ecological effects of antibiotics on natural ecosystems: a review (26)	390	130	2018	Microchem J.	Italy
18	de Kraker, MEA	Mortality and Hospital Stay Associated with resistant *Staphylococcus aureus* and *Escherichia coli* bacteremia: estimating the burden of antibiotic resistance in Europe (24)	360	36	2011	PLos Med.	Netherlands
19	Meng, XZ	Usage, residue, and human health risk of antibiotics in Chinese aquaculture: a review (216)	365	91.25	2017	Environ. Pollut.	China
20	Diamadopoulos, E	Detection and fate of antibiotic resistant bacteria in wastewater treatment plants: a review (217)	351	43.88	2013	Ecotox. Environ. Safe.	Greece

TC, total citations; TCY, total citations per year.

### Analysis of ESI hot papers

ESI hot papers are papers published in the last 2 years that have a citation frequency ranked within the top 0.1% worldwide in the previous 2 months. Three ESI hot papers published in 2020 were identified (Table 7). The hottest papers in the last 2 years describe the generation and fate of antibiotics, ARB and ARGs in sewage treatment plants around the world. The second paper reviews the research progress of antimicrobial nanofiber wound dressings since 2015, especially recent advances in biohybrid dressings made from cross species. The last hot paper summarizes the physicochemical properties of 5 photothermal agents and their application in antimicrobial photothermal therapy.

**Table 7:** The hot papers of ESI in ARB research field.

**Rank**	**Corresponding authors**	**Title**	**TC**	**Publication year**	**Journal**	**Country/Region**
1	Wang, JL	Occurrence and fate of antibiotics, antibiotic resistant genes (ARGs) and antibiotic resistant bacteria (ARB) in municipal wastewater treatment plant: an overview (220)	155	2020	Sci. Total Environ.	China
2	Boccaccini, AR	Antibacterial biohybrid nanofibers for wound dressings (221)	146	2020	Acta Biomater.	Germany
3	Peng, Q	Nanomaterials-based photothermal therapy and its potentials in antibacterial treatment (44)	67	2020	J. Control. Release	China

TC, total citations.

## Latest developments

From January 2021 to 2022, 19 highly cited papers in total met the search conditions, among which 2 were hot papers. The research contents of these highly cited papers mainly focus on the three aspects as follows. Initially, there are many researches on substances and preparations that can play an antibacterial role. For example, the antibacterial mechanism of nanomaterials (222), and molecularly imprinted polymers (223), the research review of antibacterial peptides in the source, structure, clinical trials (224), etc., the mechanism of prebiotics to remove intestinal pathogens (225), as well as the activity and antibacterial mechanism of antimicrobial agents from plants (226). Secondly, there are also many studies on the existence of micro pollutants, including the distribution and concentration of antibiotic resistance genes in the environment (227), the pollution status, sources and potential risks of antibiotics in surface water (228), and the production and removal of resistant microorganisms in hospital wastewater (229). What's more, these studies also touched upon aspects of water treatment technology, such as the mechanism of action of photocatalytic removal of antibiotics and inactivated bacteria (230), the effect of ozone removal of ARB and ARGs (231), and the overview of microalgae for environmental remediation (232).

## Discussion

### Emerging research elements

According to the statistical analysis of author keywords from 2010 to 2020, new author keywords have emerged in this field. Since the new author keywords appear less frequently, which has not shown in the chart. Here only introduce the new author keywords that appear comparatively more frequently. The 2019 COVID-19 pandemic, triggered by SARS-CoV-2 (233–236), has placed a tremendous burden on both the health care system and human society (237–239). It was found that the incidence of carbapenem-resistant Enterobacteriaceae infections have rapidly increased in critically ill patients with COVID-19 (240). Surprisingly, maintaining social distance has been shown to help reduce the transmission of SARS-CoV-2 and ARB (241). In addition, polypeptides are not only potential substitutes for the treatment of ARB infection but are also effective in the treatment of COVID-19 (242). Nanoparticles are not only effective antibacterial agents but also antibacterial drug delivery carriers. Electrospinning represents a new technology for preparing nanofibers in the last 2 years, and it is very suitable for generating antibacterial nanomaterials because nanomaterials produced using this technology have a large specific surface area and controllable structure (221, 243). In the past 2 years, studies have linked machine learning with ARB identification. Compared with traditional DNA sequencing, spectral diagnostic data are analyzed by machine learning algorithms to accurately identify ARB and ARGs (244, 245). In addition, studies have applied machine learning models for the early prediction of subclinical mastitis to reduce the risk of ARB (246).

### Future research directions

It is well known that the goal of studying antibiotic resistant bacteria is to resist ARB by understanding the mechanisms of the generation, evolution as well as transmission of the antibiotic resistance, such as the implement of sewage treatment processes; to find effective methods to reduce the harm caused by antibiotic resistant bacteria to global humankind and ecosystem, such as the research and development of new antibiotics, antibiotic substitutes, adjuvants.

According to the author keywords bubble chart (Figure 6), cross-correlation graph (Figure 7) and ESI highly cited papers (Table 6), it can be found that the research on antibiotic resistance has been the first place and plays a leading role in this field for the last decade. The scope of research mainly includes the existence of antibiotic resistance in the aquatic systems (247), sewage treatment processes, and negative effects (248, 249). This may be related to the early abuse of antibiotics (250) in many countries, such as China (142, 251–254), USA (255, 256), India (257), Italy (258), and so on. It is undeniable that those studies play a significant role in the understanding of antibiotic resistance. However, some studies have pointed out that MRSA existed long before the antibiotics was used (259). Mutations in microbial metabolism can also lead to antibiotic resistance (260). This just goes to show that our understanding of antibiotic resistance is not thorough enough. Further research on the induction factors and relevant mechanisms that lead to antibiotic resistance is required in the future.

According to the ESI hot papers (Table 7), nanomaterials have been the hottest topic in this field in the last 2 years, which is closely related to their superior antibacterial properties. However, according to the author keywords bubble chart (Figure 6) and cross-correlation graph (Figure 7), it can be found that the research on antimicrobial peptides and bacteriophages has gradually increased in the last decade but has not received enough attention. Peptide-based antibiotics have been found to be effective against MDRB because bacterial resistance responds slowly to the action mode of peptide natural products (261). Encrypted peptide kills bacteria by targeting the cell membranes of pathogenic bacteria and is not susceptible to selective resistance (262). At present, research has found candidate peptide antibiotics in human intestinal flora using machine learning (263), which breaks through the path dependence on the traditional antibiotic discovery. Bacteriophages have been found in human intestines either, which are in a harmonious symbiotic relationship with intestinal flora, rather than an antagonistic mode (264). Bacteriophage related therapies are in the concern once more (265). In addition, there has been also some progress in the relationship between intestinal flora and antibiotic resistance (266), the effect of antibiotics on intestinal flora (267), the effect of vaccines on antibiotic resistance (268), and antibiotic-resistant bacterial inhibitors (269). However, these studies are not thorough enough (270, 271). Therefore, it is necessary to pay attention to the diversification of research and strengthen the research on antibiotic substitutes, human intestinal flora and adjuvants in the future.

Antibiotic resistance imposes a heavy burden on human beings. A study on the worldwide burden of antibiotic resistance (272) found that the mortality in the whole age interval caused by antibiotic resistance is the highest in the Africa. Pseudomonas aeruginosa, MRSA and other MDRB have caused a large number of deaths. This suggests that low-resource settings bear the heaviest burden, which is consistent with the statistical analysis of this study in the leading countries or regions (Table 1), leading institutions (Table 2) and leading authors (Table 4). Although countries in Africa have made some contributions in this field (273–278), the relevant research is not sufficient and is not in the leading position, the understanding of antibiotic resistant bacteria is not enough. According to the author keywords bubble chart (Figure 6), it can be found that MRSA, Pseudomonas aeruginosa and other MDRB have received more attention in recent 2 years (279). The extremely strong resistance not only causes great losses to humans, but also threatens the existing antibiotics. Studies have shown that the COVID-19 pandemic has led to overuse of antibiotics in many areas, which will aggravate the antibiotic resistance (280, 281). Therefore, every country needs to establish strict antibiotic prescription guidelines to regulate antibiotic use. However, one study has shown that reducing antibiotic prescriptions cannot stop the spread of antibiotic resistant (282). There is a gap between antibiotic stewardship in the paper and in practice (283). Even treatments that match susceptibility of pathogens may result in resistance, because the development of antibiotic resistance is essentially driven by rapid re-infection of different strains of the patient with prescription resistance (284), and they suggest that the personalized antibiotic treatment suggestions can be given by predicting the patient's past infection or history using the machine learning, thus reducing the emergence of ARB. However, ARB can circulate and transfer between humans and animals. Therefore, it is not enough to reduce the propagation of antibiotic resistance by simply managing the use of antibiotic in human beings. There is no boundary among environment, animal and human beings. The control of antibiotic resistance requires simultaneous communication and cooperation of these three fields, rather than the separation of them (285).

In conclusion, this research proposed the possible future research direction in the field of ARB by starting from the aspects of controlling the transmission of ARB and developing new antibiotics. Aspect of relevant research on new antibacterial agents: As peptide-based antibiotics have potential to defend against the ARB, many scholars are paying attention to its design and development (286–288). However, studies show that some problems occur after this kind of antibiotics are used, for example, it causes short half lives *in vivo*, protease degradation and others (289). Therefore, the research on the interaction between peptide-based antibiotics and human bodies (290, 291) and the decoration of its chemical structure (261) shall be further conducted in the future. In addition, it is inevitable for peptide-based antibiotics to become drug-resistant, despite its relatively low possibility of becoming antibiotic resistant. So, it is required to concern how to limit the drug resistance rate of new peptide-based antibiotics in the future. In the future, it is possible to research how to use bacteriophages to recover the complexity of damaged microbiota and how to use bacteriophages to operate HGT microbial genomes in microbial flora from the mutual beneficial aspect between intestinal bacteria and bacteriophages (264). Aspect of controlling the transmission of ARB: In conclusion, corresponding measures shall be taken on three aspects including humans, animals and environment to control the transmission of ARB in the future. On the aspect of humans, concerning the gap between antibiotics management and research and the actual situation (283), it is required to research the actual using condition antibiotics in humans across the world. In addition, it is equally important to reduce the use of antibiotics so as to control the generation and transmission of antibiotic resistance, especially in countries short of resources (292). Therefore, it is demanded to research the measures on how to reduce the use of antibiotics in the future, for instance, to develop relevant vaccines or hygiene system (293, 294), etc. On the aspect of environment, wastewater can transmit ARB and ARGs not only to humans, but also to the ecological environment (19). Despite the growing number of studies on sewage treatment, there is still a lack of a unified standard and program for sewage treatment. In terms of animal husbandry and aquaculture, a global policy is required to control the use of antibiotics on animals and prevent the ARB and ARGs from spreading to humans through food chains (295). What's more, we should also research how to use and manage antibiotics jointly from the three aspects of humans, animals and environment. It is possible to develop toward the direction of constructing the biological risk assessment platform (296) and electronic monitoring system (293, 297).

## Conclusions

In this study, we provided a research overview of the field of ARB. Over time, ARB have become a global threat, and an increasing amount of related research has been carried out. Both developed countries, represented by the USA, and developing countries, represented by China, have made significant contributions to this field. There are relatively few relevant studies from Africa, but antibiotic-resistant bacterial infections in Africa are of great concern (298). ARB represent an interdisciplinary research field, with most studies focused on environmental and microbial aspects. Particularly, antibiotic resistance is not only a research focus in this field but also a research hotspot. Although some progress has been made with novel antibiotics, further research is still needed (299–301). In the future, we can strengthen the financial support (302) and technical and knowledge cooperation (303) for the research and development of new antibacterial drugs (304–306), etc. In this case, bacteriocins, phage therapy, nanomaterials, human intestinal flora and machine learning have inspired hope for the treatment of ARB infection. However, further relevant studies are still needed in the future. Since 2021–2022 related publications are not included, this study provides an overview of the latest research progress in this field based on the 2021–2022 ESI highly cited papers in the field of ARB.

Certain limitations were observed in this study. For example, articles without authors keywords were not included in the analysis. In summary, this study will hopefully inspire researchers in the field of ARB and assist them in further understanding the research trends, research hotspots, and future research directions in this field. Although WOS has covered many publications, however, some publications from database such as Scopus, PubMed, may not be included in this study.

## Author contributions

YW and QZ contributed to the conception and design of the study and wrote the first draft of the manuscript. QZ organized the database and performed the statistical analysis. GS and DD reviewed and edited the manuscript. GS and ZD provided financial support. All authors contributed to manuscript revision, read, and approved the submitted version.

## Funding

This research was funded by Science Technology Department of Zhejiang Province (No. 2022C25007).

## Conflict of interest

The authors declare that the research was conducted in the absence of any commercial or financial relationships that could be construed as a potential conflict of interest.

## Publisher's note

All claims expressed in this article are solely those of the authors and do not necessarily represent those of their affiliated organizations, or those of the publisher, the editors and the reviewers. Any product that may be evaluated in this article, or claim that may be made by its manufacturer, is not guaranteed or endorsed by the publisher.
